# CHA_2_DS_2_-VASc score as a predictor of clinical outcomes in hospitalized patients with and without chronic kidney disease

**DOI:** 10.1007/s40620-023-01805-7

**Published:** 2023-11-08

**Authors:** Antonietta Gigante, Giovanni Imbimbo, Martina Andreini, Marco Proietti, Mariangela Palladino, Alessio Molfino, Danilo Alunni Fegatelli, Maurizio Muscaritoli

**Affiliations:** 1 Department of Translational and Precision Medicine, Sapienza University of Rome, Rome, Italy; 2 Division of Subacute Care, IRCCS Istituti Clinici Scientifici Maugeri, Milan, Italy; 3 Department of Clinical Sciences and Community Health, University of Milan, Milan, Italy; 4 Department of Public Health and Infectious Diseases, Sapienza University, Rome, Italy

**Keywords:** CHA_2_DS_2_-VASc score, Chronic kidney disease, Internal medicine, mortality, length of stay

## Abstract

**Background:**

High CHA_2_DS_2_-VASc score (Congestive heart failure, Hypertension, Age > 75 years, Diabetes mellitus, prior Stroke or transient ischemic attack or thromboembolism, Vascular disease, Age 65–74 and Sex category) was associated with adverse clinical outcomes in different settings.

The aim of the present study was to evaluate the association between CHA_2_DS_2_-VASc score and R_2_CHA_2_DS_2_-VASc score (which includes renal impairment) with in-hospital mortality and length of hospital stay in patients hospitalized in an internal medicine ward.

**Methods:**

We enrolled 983 consecutive patients admitted during 3 years in an internal medicine ward. R_2_CHA_2_DS_2_-VASc score was calculated by adding 2 points to CHA_2_DS_2_-VASc for the presence of chronic kidney disease (CKD), defined according to K-DOQI. The primary outcome was a composite of all-cause mortality and length of hospital stay > 10 days.

**Results:**

Patients with CKD stages 3–5 presented with increased CHA_2_DS_2_-VASc vs stages 1–2 (*p* < 0.001). The composite outcome occurred in 47.3% of inpatients. Multivariable linear logistic regression analyses adjusted for presence of infectious diseases and cancer, with the occurrence of composite outcome showed an adjusted OR of 1.349 (95% CI 1.248–1.462) and 1.254 (95% CI 1.179–1.336) for CHA_2_DS_2_-VASc and R_2_CHA_2_DS_2_-VASc scores, respectively. No differences were found in the association between CHA_2_DS_2_-VASc and R_2_CHA_2_DS_2_-VASc scores with the composite outcome (AUC 0.631 vs 0.630), and furthermore, adding the presence/absence of infectious diseases during hospitalization and positive cancer history to the models increased the AUC (0.667 and 0.663).

**Conclusions:**

Incrementally higher CHA_2_DS_2_-VASc score is associated with increased length of hospital stay and mortality in patients hospitalized in an internal medicine ward, regardless of the presence of CKD.

**Graphical abstract:**

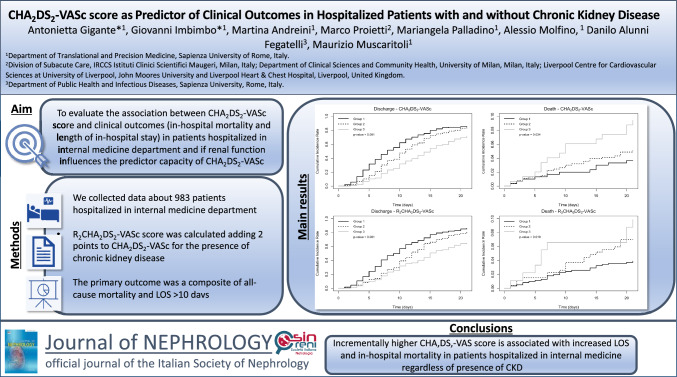

## Introduction

Chronic kidney disease (CKD) is an important cause of mortality and morbidity, and continues to increase worldwide with an estimated prevalence of 13.4% [1]. Chronic kidney disease is defined as abnormalities of kidney structure or function, present for > 3 months [2]; it is highly prevalent in older patients and impacts drug prescription [3]. It has been demonstrated that the presence of CKD can predict the development of fatal and non-fatal cardiovascular events [4].

In a recent study in hospitalized patients with atrial fibrillation with 2.7 years of follow-up, the presence of CKD was independently associated with reduced survival, and an estimated Glomerular Filtration Rate (eGFR) < 50 ml/min/1.73 m^2^ was associated with worse prognosis [5].

The Congestive heart failure, Hypertension, Age > 75 years, Diabetes mellitus, prior Stroke or transient ischemic attack or thromboembolism, Vascular disease, Age 65–74 and Sex category (CHA_2_DS_2_-VASc) is a simple score that was initially used for stroke risk stratification in atrial fibrillation patients [6]. However, recently the CHA_2_DS_2_-VASc score was also used in different settings other than atrial fibrillation, such as end-stage kidney disease (ESKD) or hemodialysis to predict other clinical outcomes, including mortality in non-atrial fibrillation patients [7–9]. Patients with CKD are notably exposed to more cardiovascular risk factors than non-CKD patients and the use of the CHA_2_DS_2_-VASc score in this setting as a predictor of clinical outcomes is poorly explored. In addition, R_2_CHA_2_DS_2_-VASc is a modified form of CHA_2_DS_2_-VASc, created by adding 2 points in case of impaired renal function [8, 9].

The aim of the present study was to evaluate the association between CHA_2_DS_2_-VASc score and R_2_CHA_2_DS_2_-VASc with in-hospital mortality and length of hospital stay in patients hospitalized in an internal medicine ward.

## Methods

### Study population

We conducted a cross-sectional study in a cohort of patients admitted to the Department of Internal Medicine, Sapienza University of Rome, Italy, over 3 consecutive years before the COVID-19 pandemic. Exclusion criteria were: age < 18 years and presence of acute kidney injury (AKI) at hospital admission. The study was conducted in accordance with the Declaration of Helsinki. All patients provided informed consent. The study project was approved by the Local Ethics Committee.

Presence and stage of CKD were characterized according to K-DOQI (Kidney Disease Outcomes Quality Initiatives) guidelines [2].

Renal function was defined by eGFR, considering creatinine values at hospital admission of non-AKI patients. To estimate eGFR, we used the new Chronic Kidney Disease Epidemiology Collaboration (CKD-EPI) equation expressed as a single equation, using the serum creatinine value (Scr) as follows: eGFRcr = 142 × min(Scr/*κ*, 1)*α* × max(Scr/*κ*, 1) − 1.200 × 0.9938Age × 1.012 [if female]; where Scr is standardized serum creatinine in mg/dL, *κ* is 0.7 for females or 0.9 for males, *α* is − 0.241 for females or − 0.302 for males, min(Scr/*κ*, 1) is the minimum of Scr/*κ* or 1.0, max(Scr/*κ*, 1) is the maximum of Scr/*κ* or 1.0 [10]. We collected clinical information including personal data, primary diagnosis, comorbidities, biochemical analyses, length of hospital stay and death from the patients’ clinical records. We recorded prevalence of cardiovascular risk factors, such as diabetes, dyslipidemia, hypertension, history of stroke, chronic heart failure and previous acute coronary syndrome.

### CHA_2_DS_2_-VASc and R_2_CHA_2_DS_2_-VASc calculation

The CHA_2_DS_2_-VASc score was calculated at admission to hospital for all patients by evaluating the following parameters [6]: presence/history of congestive heart failure, hypertension, age, diabetes mellitus, stroke or transient ischemic attack, vascular disease, sex. We also calculated the R_2_CHA_2_DS_2_-VASc score by adding 2 points to the CHA_2_DS_2_-VASc score for patients with eGFR < 60 ml/min/1.73 m^2^ [11].

#### Outcome definition

As clinical outcomes, we considered the length of hospital stay from admission to our department of internal medicine and the in-hospital mortality. The primary outcome was a composite of all-cause mortality and length of hospital stay > 10 days. As the secondary outcome, we evaluated the individual items of the composite outcome.

### Statistical analysis

Population characteristics were reported according to the presence/absence of CKD. Numerical variables were expressed as mean (standard deviation) and median (interquartile range). Categorical variables were presented as absolute frequencies (percentages). Statistical differences in CHA_2_DS_2_-VASc score among CKD stage groups were represented through boxplots and evaluated using Kruskal–Wallis test followed by Dunn’s post hoc test for multiple comparisons.

Logistic regression analysis and receiver operating characteristic (ROC) curve analysis were used to evaluate the prognostic performance of the CHA_2_DS_2_-VASc score of the composite endpoint.

Competing risk analysis was used to take into account death as a competing event and to determine differences in length of hospital stay giving estimates of discharge rates.

The analysis was performed using the statistical software R (version 4.2.0). A significance level of 0.05 was used for all tests.

## Results

### Patients’ characteristics

We collected data concerning 983 inpatients with a mean age of 66.9 years ± 16.4 (females 42.4%) admitted to the internal medicine ward during the study period. Patients’ characteristics are summarized in Table 1. In our cohort, an eGFR < 60 ml/min/1.73 m^2^ was present in 215/983 (21.9%) patients, and distribution across the stages of the disease were: stage 3, 158 (16.1%); stage 4, 36 (3.7%); stage 5, 21 (2.1%). Nine patients with stage 5 CKD and one with stage 4 received hemodialysis. At admission, mean creatinine was 1.1 ± 0.9, with a mean eGFR (ml/min/1.73 m^2^) of 81.7 ± 27.6.

**Table 1:** Patients’ characteristics

Parameters	All patients (*N* = 983)	Stages 1–2 CKD (*N* = 768)	Stages 3-4-5 CKD (*N* = 215)	*p*-value
Sex (female)	417 (42.4)	317 (41.3)	100 (46.5)	0.195
Age, years	66.9 ± 16.4	64.6 ± 16.8	75.1 ± 11.7	< 0.001
Creatinine at admission, mg/dl	1.1 ± 0.9	0.8 ± 0.2	2.0 ± 1.6	< 0.001
Creatinine at admission, mg/dl	0.9 (0.7–1.1)	0.8 (0.6–0.9)	1.5 (1.2–2.1)	< 0.001
eGFR (CKD-EPI), ml/min/1.73 m^2^	81.7 ± 27.6	93.2 ± 17.2	40.3 ± 15.3	< 0.001
eGFR (CKD-EPI), ml/min/1.73 m^2^	87.4 (63.6–100.9)	93.5 (80.4–104.7)	44.9 (29.1–51.9)	< 0.001
CHA_2_DS_2_-VASc score	3 (1; 4)	2 (1; 3)	4 (3; 5)	< 0.001
R_2_CHA_2_DS_2_-VASc score	3 (1; 4)	2 (1; 3)	6 (5; 7)	< 0.001
Length of hospital stay, days	10 (6; 15)	10 (6; 15)	12 (7; 19)	< 0.001
In-hospital mortality	62 (6.3)	45 (5.9)	17 (7.9)	0.351
*Comorbidities*				
Atrial fibrillation, *n* (%)	125 (12.7)	74 (9.6)	51 (23.7)	< 0.001
Diabetes, *n* (%)	35 (3.6)	12 (1.6)	23 (10.7)	< 0.001
Hypertension, *n* (%)	492 (50.1)	337 (43.9)	155 (72.1)	< 0.001
Coronary artery disease, *n* (%)	159 (16.2)	94 (12.2)	65 (30.2)	< 0.001
COPD, *n* (%)	118 (12)	81 (10.5)	37 (17.2)	0.011
OSAS, *n* (%)	23 (2.3)	17 (2.2)	6 (2.8)	0.803
Stroke or TIA, *n* (%)	76 (7.7)	35 (4.6)	41 (19.2)	< 0.001
Cancer, *n* (%)	177 (18.0)	134 (17.4)	43 (20.0)	0.447
Liver disease, *n* (%)	62 (6.3)	43 (5.6)	19 (8.9)	0.109
Heart failure, *n* (%)	90 (9.2)	58 (7.6)	32 (14.9)	0.002
Dyslipidemia, *n* (%)	39 (4.0)	26 (3.4)	13 (6.0)	0.117
Peripheral arteriopathy	82 (8.3)	19 (2.5)	63 (29.3)	< 0.001
Nephrotic syndrome, *n* (%)	20 (2.0)	13 (1.7)	7 (3.3)	0.245
*Events during admission*				
Infectious diseases (all), *n* (%)	159 (16.2)	134 (17.4)	25 (11.6)	0.052
Pneumonia, *n* (%)	72 (7.3)	63 (8.2)	9 (4.2)	0.064
Urinary tract infection, *n* (%)	20 (2.0)	13 (1.7)	7 (3.3)	0.245
Sepsis, *n* (%)	10 (1.0)	7 (0.9)	3 (1.4)	0.810
Other infections, *n* (%)	73 (7.4)	62 (8.1)	11 (5.1)	0.189
Pulmonary embolism, *n* (%)	13 (1.3)	12 (1.6)	1 (0.5)	0.364
Deep vein thrombosis, *n* (%)	14 (1.4)	12 (1.6)	2 (0.9)	0.714

Numerical variables are shown as mean (standard deviation) or median (interquartile range), according to the variable distribution

*COPD* chronic obstructive pulmonary disease, *OSAS* Obstructive Sleep Apnea Syndrome

Differences in main demographic and clinical parameters between patients according to renal function are described in Table 1.

The most prevalent comorbidity was hypertension, that was present in approximately half of the participants, followed by diabetes (22.1%), CKD and ischemic cardiovascular disease (16.2%). History of cancer was present in 177 (18.0%) participants. During hospitalization, we diagnosed infectious diseases, including urinary tract infections, pneumonia, cellulitis, and viral and parasitic infections in 159 inpatients (16.2%), as shown in Table 1.

#### Differences in CHA_2_DS_2_-VASc score among CKD and non-CKD patients

We calculated CHA_2_DS_2_-VASc and R_2_CHA_2_DS_2_-VASc scores in all the participants included in the study. The median of CHA_2_DS_2_-VASc and R_2_CHA_2_DS_2_-VASc was 3 (IQR 1–4).

**Fig. 1:**
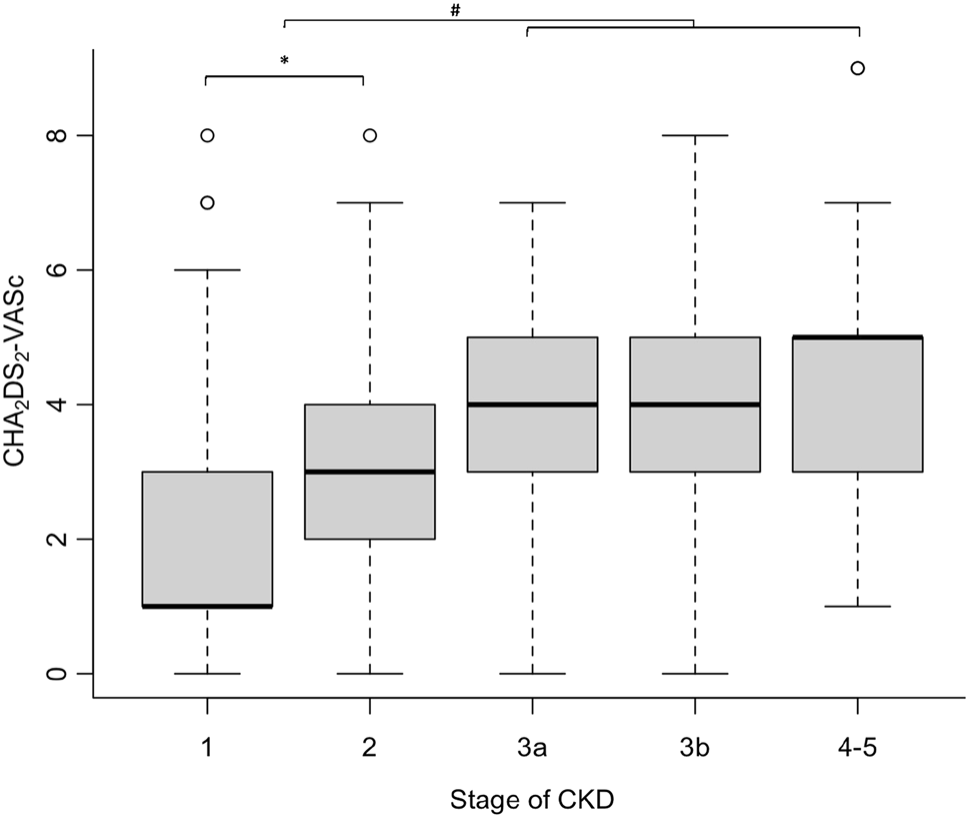
Differences in CHA2DS2-VASc score according to the stage of CKD. *Difference between stage 1 vs. stage 2 (*p* < 0.001); ^#^Differences between stage 1 and 2 vs stage 3a, 3b, 4–5 (*p* < 0.01). *CKD* chronic kidney disease

Patients with stages 3-4-5 (eGFR < 60 ml/min/1.73 m^2^) presented with an increased CHA_2_DS_2_-VASc score with respect to stages 1 and 2 (eGFR ≥ 60 ml/min/1.73 m^2^) (*p* < 0.001) (Fig. 1). Moreover, no significant differences were observed in patients with eGFR < 60 ml/min/1.73 m^2^ according to the stage of the disease for CHA_2_DS_2_-VASc score (Fig. 1). All the items of CHA_2_DS_2_-VASc were more significant in CKD patients with respect to those without CKD, except for sex (Table 1).

#### Association of CHA_2_DS_2_-VASc and R_2_CHA_2_DS_2_-VASc with length of hospital stay and survival in all patients

The median of length of hospital stay was higher in patients with low eGFR with respect to non-CKD patients [12 (IQR 7–19) vs. 10 (IQR 6–15)], (*p* < 0.001).

We documented a total of 62 (6.3%) deaths among all the participants; 45 (5.9%) in non-CKD patients and 17 (7.9%) among CKD patients (*p* = 0.351). The composite outcome occurred in 465 (47.3%) patients. Multivariable linear logistic regression analyses were used to evaluate the association between CHA_2_DS_2_-VASc and R_2_CHA_2_DS_2_-VASc score, adjusted by the presence/absence of infectious diseases and cancer history, with the occurrence of composite outcome.

**Fig. 2:**
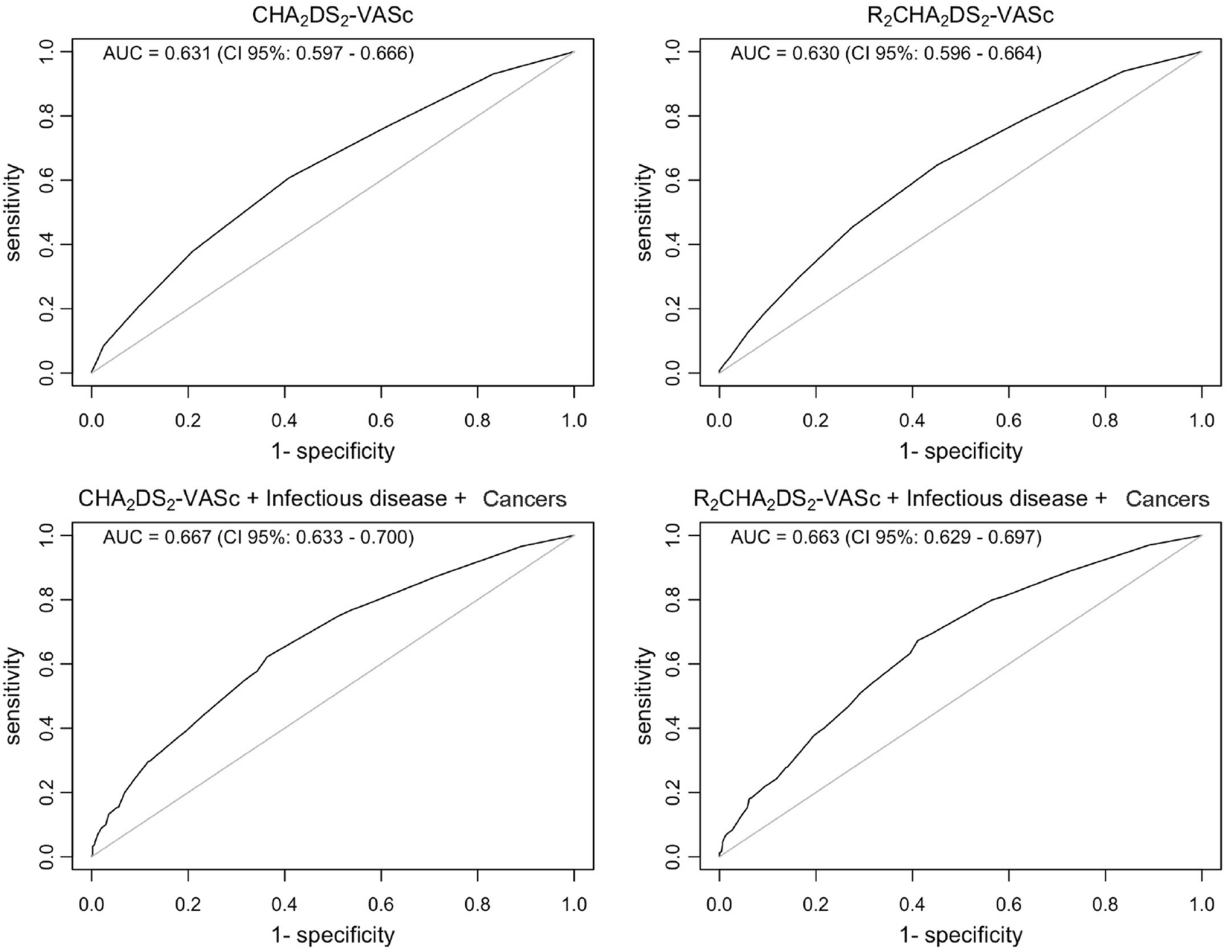
Prognostic receiver operating characteristic (ROC) curves evaluating the association of CHA_2_DS_2_-VASc and R_2_CHA_2_DS_2_-VASc score, in addition to the presence/absence of infectious diseases and cancer history, with the occurrence of composite outcome

The adjusted ORs were 1.349 (95% CI 1.248–1.462) and 1.254 (95% CI 1.179–1.336) for CHA_2_DS_2_-VASc and R_2_CHA_2_DS_2_-VASc scores, respectively (*p* < 0.001). The corresponding prognostic ROC curves are shown in Fig. 2. Importantly, no differences were present in the association between CHA_2_DS_2_-VASc and R_2_CHA_2_DS_2_-VASc scores with the composite outcome (AUC 0.631 vs 0.630, respectively). Prognostic ROC curves, designed by adding the presence/absence of infectious diseases during hospitalization and positive cancer history to the models mentioned above, showed increased AUC with respect to CHA_2_DS_2_-VASc and R_2_CHA_2_DS_2_-VASc score alone (AUC 0.667 and AUC 0.663, respectively).

**Fig. 3:**
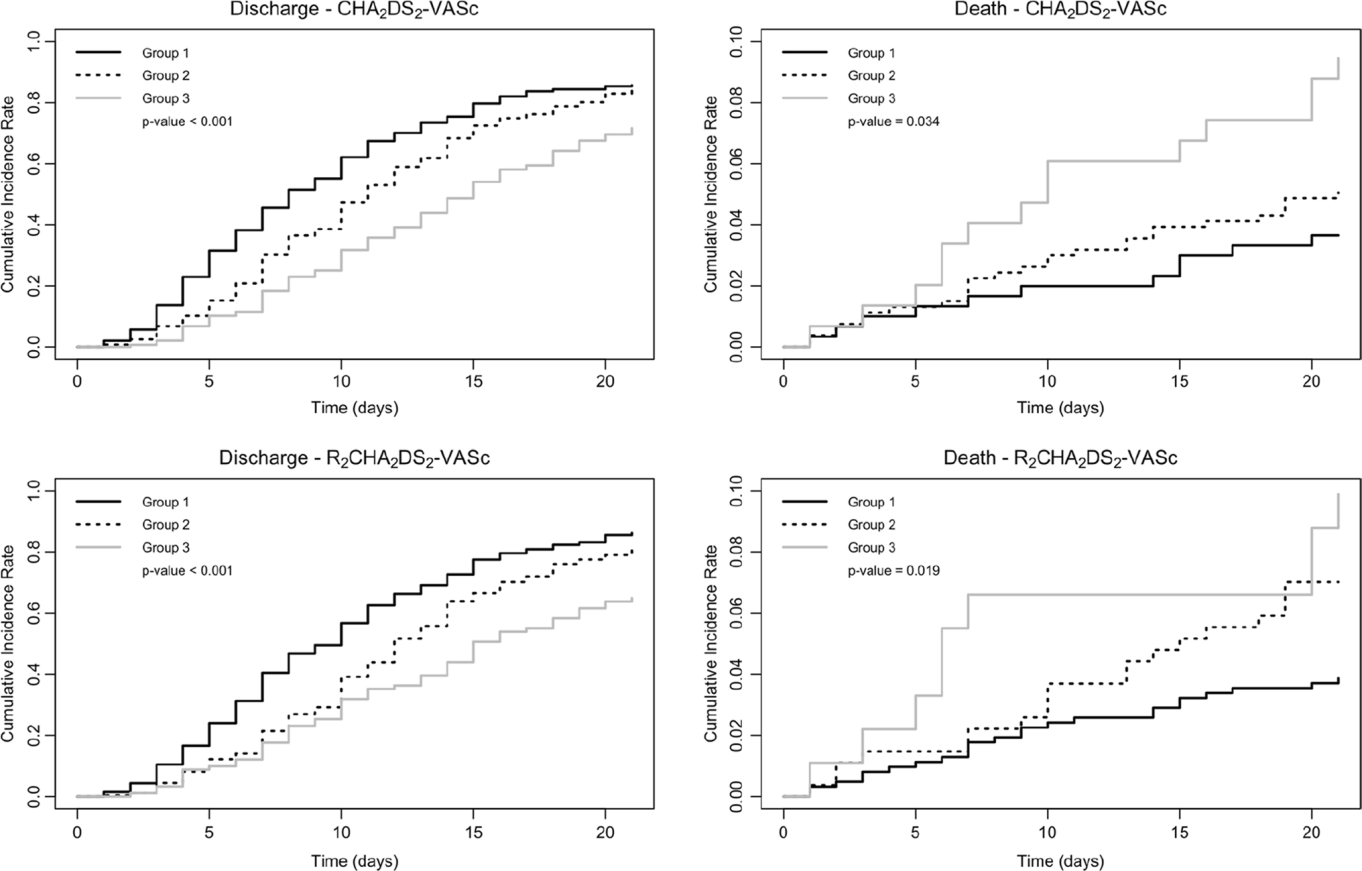
Cumulative incidence rates of mortality and discharge for CHA_2_DS_2_-VASc score, group 1 = 0–1; group 2 = 2–4; group 3 = 5–9. For R_2_CHA_2_DS_2_-VASc score, group 1 = 0–3; group 2 = 4–6; group 3 = 7–11

We then analyzed the individual clinical outcomes by performing a competing risk analysis providing estimates of the cumulative incidence rates of mortality and discharge (Fig. 3). We divided patients into 3 groups according to the CHA_2_DS_2_-VASc score (Fig. 3). Groups 1 and 3 represented patients at low and high-risk of mortality, respectively, as suggested by previous studies [12, 13].

Patients in CHA_2_DS_2_-VASc groups 2 and 3, with median length of hospital stay of 11 (IQR 8–17) and 15 (IQR 9–21) days, respectively, showed an increased length of hospital stay with respect to group 1 [8 (IQR 5–14)] (*p* < 0.001).

Also, patients in CHA_2_DS_2_-VASc groups 2 and 3 died earlier during their hospitalization with respect to those in CHA_2_DS_2_-VASc group 1 (*p* = 0.034) (see Fig. 3).

However, R_2_CHA_2_DS_2_-VASc score groups presented no differences with regard to length of hospital stay and mortality compared to data obtained using CHA_2_DS_2_-VASc score (see Fig. 3).

## Discussion

The CHA_2_DS_2_-VASc score has been widely used to predict the risk of stroke in patients with atrial fibrillation, however in this cross-sectional study we aimed at investigating the clinical use of the CHA_2_DS_2_-VASc score as a predictor of length of hospital stay and mortality in patients hospitalized in our internal medicine department by focusing on the presence of CKD.

In agreement with several studies [14–16], we observed that inpatients with high CHA_2_DS_2_-VASc scores presented a significant increase in length of hospital stay and in-hospital mortality. The predictive role of CHA_2_DS_2_-VASc and mortality/length of hospital stay has been evaluated in different diseases such as myocardial infarction [14], acute pulmonary embolism [15] and in patients hospitalized with COVID-19 [16].

However, adding the presence of CKD to this score (R_2_CHA_2_DS_2_-VASc score) did not ameliorate the ability of the CHA_2_DS_2_-VASc score to predict the outcomes. Previous studies investigated the use of CHA_2_DS_2_-VASc score and R_2_CHA_2_DS_2_-VASc score for the prediction of clinical outcomes, showing the validity of these tools in identifying patients with poor prognosis [17–19]. Interestingly, Harb et al. [12] showed that in a large cohort of out-patients, regardless of the presence or absence of atrial fibrillation, high CHA_2_DS_2_-VASc score was associated with higher all-cause mortality. Specifically, in outpatients with CKD, CHA_2_DS_2_-VASc score predicts all cause and cardiovascular mortality [20]. To the best of our knowledge, this is one of the few studies that describes the use of CHA_2_DS_2_-VASc score in inpatients for assessing the presence of CKD, showing that this tool may be clinically useful in risk stratification. The complex relationship between kidney and cardiovascular disease has been extensively investigated, although some pathophysiological mechanisms are still to be understood [21]. It is well known that CKD is associated with accelerated cardiovascular disease risk and a higher cardiovascular event rate [22]. We believe that the high cardiovascular burden associated with CKD is clearly identified by the high CHA_2_DS_2_-VASc score in our patients. The presence of high CHA_2_DS_2_-VASc in CKD patients is probably the reason for the lack of difference between CHA_2_DS_2_-VASc and R_2_CHA_2_DS_2_-VASc in the ability to predict clinical outcome.

Furthermore, R_2_CHA_2_DS_2_-VASc did not add significance in predicting clinical outcome, unlike other scores such as the Sequential Organ Failure Assessment (SOFA) scale (primarily created for mortality prediction in septic patients [23]), which is based on several parameters reflecting multi-organ failure including renal function. As previously stated, CKD patients showed higher cardiovascular event rates [22] and the creatinine concentration itself is an independent risk factor for mortality [24]. Several studies showed that very slight changes in serum creatinine during hospitalization are associated with an independent, higher risk of death [25, 26].

However, for patients admitted to the internal medicine ward, other relevant parameters not included in CHA_2_DS_2_-VASc and R_2_CHA_2_DS_2_-VASc may significantly affect the prognosis. In our cohort we observed a high prevalence of patients with cancer, infections and liver diseases in line with the usual characteristics of patients admitted to an internal medicine ward. In an observational study conducted on 635 inpatients, over 40% of patients had a possible infection, while 15% had sepsis [27]. In our previous study involving 1087 patients admitted to an internal medicine ward, we found that infectious diseases, particularly in the elderly, played a key role as trigger factors for the development of cardiorenal syndrome, thus increasing length of hospital stay [28]. Moreover, in a REPOSI registry, among 6047 patients enrolled, 2991 (49.5%) were diagnosed with at least one infection [29].

Admissions of patients with cancer are distributed across different medical units, with 44% being concentrated in the medical oncology unit and 12% in internal medicine wards [30]. Cancer diseases require frequent admission and long hospital stay [30]. To evaluate the impact of these factors in predicting mortality and length of hospital stay, we added the presence of cancer history and infectious diseases to the model, showing increased sensitivity in the detection of the composite outcome, as described in the ROC curves. Moreover, our multivariate Cox regression analysis showed that both the CHA_2_DS_2_-VASc, presence of infectious diseases and cancer are independent predictors of mortality and increased length of hospital stay.

The cross-sectional nature of our study represents the first and intrinsic limitation; second, our population was enrolled in a single center, third, specific data on cardiovascular assessment were missing. Future epidemiologic studies are needed to confirm these data on a larger scale and to assess their clinical relevance in everyday practice.

## Conclusions

Applying the CHA_2_DS_2_-VASc score in CKD patients hospitalized in an internal medicine ward may help predict mortality and length of hospital stay. The prognostic value of the CHA_2_DS_2_-VASc score significantly increases considering the presence of infectious diseases and cancer. The lack of a predictive improvement of the modified formula adding kidney function impairment (R_2_CHA_2_DS_2_-VASc) is likely associated with the increased prevalence of cardiovascular comorbidities in patients with CKD.

## Data Availability

The datasets generated during and/or analysed during the current study are available from the corresponding author on reasonable request.
